# SARS-CoV-2 specific cellular response following COVID-19 vaccination in patients with chronic lymphocytic leukemia

**DOI:** 10.1038/s41375-021-01500-1

**Published:** 2021-12-22

**Authors:** Sibylle C. Mellinghoff, Sandra Robrecht, Leonie Mayer, Leonie M. Weskamm, Christine Dahlke, Henning Gruell, Kanika Vanshylla, Hans A. Schlösser, Martin Thelen, Anna-Maria Fink, Kirsten Fischer, Florian Klein, Marylyn M. Addo, Barbara Eichhorst, Michael Hallek, Petra Langerbeins

**Affiliations:** 1 Faculty of Medicine and University Hospital of Cologne, Department I of Internal Medicine, Centre for Integrated Oncology Aachen Bonn Cologne Düsseldorf (CIO ABCD), German CLL Group (GCLLSG), University of Cologne, Cologne, Germany; 2 German Centre for Infection Research (DZIF), partner site Bonn-Cologne, Cologne, Germany; 3 Department of Clinical Immunology of Infectious Diseases, Bernhard Nocht Institute for Tropical Medicine, Hamburg, Germany; 4 Division of Infectious Diseases, First Department of Medicine, University Medical Centre Hamburg-Eppendorf, Hamburg, Germany; 5 German Centre for Infection Research (DZIF), Partner Site Hamburg-Lübeck-Borstel-Riems, Hamburg, Germany; 6 Institute of Virology, Faculty of Medicine and University Hospital Cologne, University of Cologne, Cologne, Germany; 7 Centre for Molecular Medicine Cologne, University of Cologne, Faculty of Medicine and University Hospital Cologne, Cologne, Germany

**Keywords:** Immunological disorders, Immunological disorders

Chatzikonstantinou et al. [1] conducted a large follow-up analysis of COVID-19 in patients with chronic lymphocytic leukemia (CLL) and confirmed a high mortality rate, especially in patients with older age, comorbidity and previous CLL-treatment. The results emphasize the importance of prevention and mitigation of COVID-19 by vaccination, especially in patients with hematological malignancies. The COVID-19 vaccine-induced immunity is mediated by the interaction of both, humoral and cellular components [2, 3]. While several studies have confirmed low humoral immunogenicity in CLL patients [4–7], very few describe cellular responses to determine immunogenicity and report reduced T cell response [8]. In this prospective cohort study, we hence investigated cellular immunogenicity and the interplay with humoral immunogenicity following COVID-19 vaccination in SLL/CLL patients as compared with healthy controls (HC).

Blood samples of CLL registry (NCT02863692) patients were centrally evaluated after full COVID-19 vaccination. In total, 21/23 patients were included in the analyses (samples missing in 2/23). Vaccinated healthcare workers served as HC cohort (*n* = 12). Both studies were approved by the local ethics committee.

Patient and disease characteristics and vaccination schedules are summarized in Table 1 and Supplemental Table 1. Patient blood samples were collected at a median of 47 (range 19–94 days) and HC at a median of 35 (range 32–38) days after the second vaccination.

**Table 1:** Patients baseline characteristics and disease characteristics in the overall cohort and by subgroups.

Parameters N (%)	Patients with CLL (*N* = 23)				
	Overall cohort	Humoral response negative T cell response negative	Humoral response negative, T cell response positive	Humoral response positive, T cell response negative	Humoral response positive, T cell response positive
**Overall COVID-19 vaccine immune response**		8 (38.1)^a^	5 (23.8)^a^	5 (23.8)^a^	3 (14.3)^a^
**Age, median (range)** (years)	70 (46–79)	70.5 (48–79)	71.0 (53–79)	74.0 (62–77)	59.0 (49–62)
**Age group** (years)					
>65	13 (56.5)	6 (75.0)	3 (60.0)	4 (80.0)	0 (0.0)
>70	11 (47.8)	4 (50.0)	3 (60.0)	4 (80.0)	0 (0.0)
**Male sex**	20 (87)	6 (75.0)	4 (80.0)	5 (100.0)	3 (100.0)
**Disease / treatment status**					
Treatment-naïve	1 (4.3)	0 (0.0)	0 (0.0)	1 (20.0)	0 (0.0)
Previously treated	22 (95.7)	8 (100.0)	5 (100.0)	4 (80.0)	3 (100.0)
**Treatment prior vaccination**	22 (95.7)				
Line of treatment, median (range)	2 (1–8)	2 (1–8)	3 (2–5)	2 (1–5)	2 (1–2)
1^st^ line	6 (27.3)	2 (25.0)	0 (0.0)	2 (50.0)	1 (33.3)
>1^st^ line	16 (72.7)	6 (75.0)	5 (100.0)	2 (40.0)	2 (66.7)
Treatment < 12 months prior vaccination	9 (40.9)	3 (37.5)	4 (80.0)	1 (25.0)	0 (0.0)
without anti CD20^b^	2 (9.1)	0 (0.0)	1 (20.0)	1 (20.0)	0 (0.0)
with anti CD20^c^	7 (31.8)	3 (37.5)	3 (60.0)	0 (0.0)	0 (0.0)
**Type according to hierarchical model**^d^	21 (91.3)				
del(17p)	4 (19.0)	3 (37.5)	1 (20.0)	0 (0.0)	0 (0.0)
del(11q)	5 (23.8)	1 (12.5)	1 (20.0)	1 (33.3)	2 (66.7)
Trisomy 12	4 (19.0)	1 (12.5)	0 (0.0)	0 (0.0)	1 (33.3)
No abnormalities	1 (4.8)	0 (0.0)	0 (0.0)	1 (33.3)	0 (0.0)
del(13q) [single]	7 (33.3)	3 (37.5)	3 (60.0)	1 (33.3)	0 (0.0)
**IGHV mutational status**	18 (78.3)				
Unmutated	13 (72.2)	6 (75.0)	2 (66.7)	2 (66.7)	2 (100.0)
Mutated	5 (27.8)	2 (25.0)	1 (33.3)	1 (33.3)	0 (0.0)
**TP53 mutational status**	19 (82.6)				
Mutated	2 (10.5)	5 (71.4)	4 (100.0)	3 (100.0)	3 (100.0)
Unmutated	17 (89.5)	2 (28.6)	0 (0.0)	0 (0.0)	0 (0.0)

^a^Humoral and T cell response measured in 21/23 patients.

^b^Acalabrutinib, Ibrutinib.

^c^Obinutuzumab, Obinutuzumab/Venetoclax, Acalabrutinib/Obinutuzumab, Acalabrutinib/Obinutuzumab/Venetoclax.

^d^Cytogenetic subgroups were determined according to the hierarchical model of Döhner et al. [11].

SARS-CoV-2 receptor-binding domain (RBD) specific IgG antibodies, determined using Alinity ci SARS-CoV-2 IgG II Quant assay (Abbott), were detectable in 8/21 (38.1%) patients with SLL/CLL and 100% of HC (*p* = 0.001; Fig. 1A, B). Neutralizing activity, determined by using heat-inactivated serum in a lentiviral-based pseudovirus neutralization assay against Wu-01 strain of SARS-CoV-2, was observed in serum samples from all HC (GeoMean ID_50_ 409) (Fig. 1C). No neutralizing activity (ID_50_ < 10) was detectable in the majority of CLL patients (14/21, 67%, 0), including all seronegative individuals. However, CLL patients with detectable activity (7/21, 30%) had a response that was comparable to HC (ID_50_ 523, *p* = 0.9).

**Fig. 1:**
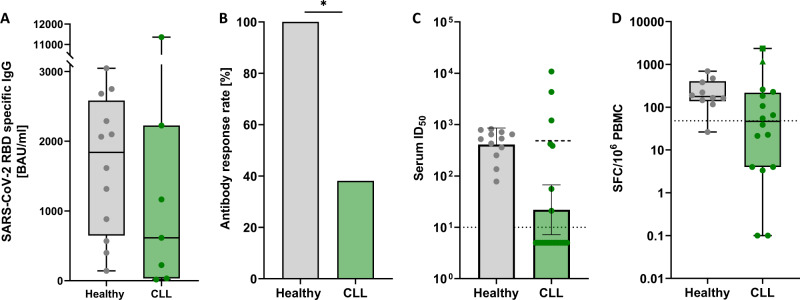
Humoral and T cell immune responses after COVID-19 vaccination. **A** SARS-CoV-2 RBD specific IgG in CLL patients (median 889.9 BAU/ml, IQR 80.2-2127.4, for responders) and healthy controls (median 1839.8 BAU/ml, IQR 647.0-2583.4) measured by ELISA. **B** Antibody response rate in CLL patients and healthy volunteers. *p = 0.001. **C** Serum neutralizing activity (50% inhibitory serum dilution) determined in a pseudovirus neutralizing assay against the Wu-01 pseudovirus strain. Bars indicating geometric mean ID50 with 95% confidence intervals. A dashed line indicates limit of detection [10]. Samples with no detectable neutralization (ID_50_ < 10) were plotted with an arbitrary ID_50_ of 5 for graphical representation. Dashed line in the CLL group shows geometric mean ID50 for individuals with a detectable neutralizing response. **D** Interferon-y T cell ELISpot response in CLL patients and HC. Shown values are mean spots in peptide-stimulated wells minus background in negative control wells. Error bars represent median ± interquartile range. The dotted line indicates the positive threshold of 48 SFC/10^6^ PBMC. Samples were acquired 35 days after the second vaccination in HC and at a median of 47 days after second vaccination in CLL patients. Two patients had much higher correlated of T cell immunity after vaccination: One was vaccinated thrice and one was the only patient of the entire cohort that had received heterologous prime-boost immunization with BNT162b and ChAdOx1. BNT BNT162b, ChAd ChAdOx1, HC Healthy Control.

Peripheral blood mononuclear cells (PBMCs) were used for SARS-CoV-2 spike-specific T cells (Human IFNy ELISpot^PLUS^ [ALP] kit [Mabtech]) and B cells (IgG ELISpot) analyses. Results are reported as spot-forming cells (SFC) per million PBMCs. T cell responses to SARS-CoV-2 peptide pool ([15-mers overlapping by 11 amino acids] spanning the entire spike protein) were considered positive if higher than twice the median response of pre-pandemic HC (48 SFC/10^6^). The median number of SARS-CoV-2 specific T cells was 21.3. SFC (interquartile range [IQR] 0.0–145.0) for CLL patients as compared with 177.3 SFC [IQR 138.0–403.3] in HC (*p* = 0.008; Fig. 1D). While 8/21 (38.1%) CLL patients had a SARS-CoV-2 spike-specific T cell response measurable above cut-off, 90% of HC mounted a response (*p* = 0.009).

SARS-CoV-2 S1/2-specific antibody-secreting cells (ASC) were analyzed in 14/21 (66.7%) SLL/CLL patients. The cut-off value for positive responses were defined as the mean plus two standard deviations of the responses observed in pre-pandemic HC (62 SFC/10^6^). Overall, 1/14 SLL/CLL patients (7.1%) had detectable S-specific ASC (138 SFC) as compared with 100% in HC (median 193 SFC, range 89-464 SFC). The SARS-CoV-2 specific IgG titer of the ASC responding patient was with 11 360 BAU/ml the highest within the group of CLL patients. Looking at total IgG-secreting B cells, 13 patients without S-specific ASC did neither show any IgG-secreting B cells. Spots were too faint to be counted or detected at numbers below the cut-off.

In a descriptive analysis (Table 1 and Supplemental Table 2 and 3), potential variables to be associated with humoral and T cell responses were investigated. While 3/21 (14.3%) of patients had both a humoral and a T cellular response, eight patients (38.1%) were double negative and a discordant response, defined by detection of either T cellular or humoral immune response to vaccination was found in most patients (10/21, 47.6%).

In conclusion, humoral and cellular immunogenicity following COVID-19 vaccination was significantly impaired in patients with SLL/CLL as described previously. SARS-CoV-2 specific antibodies and T cells were detectable in 38.1% each. In the majority of seroconverted patients, SARS-CoV-2 neutralizing serum activity of diverse magnitude was detectable indicating functionality of antibodies if at all mounted. While less than 15% of patients had both a humoral and cellular response, most patients showed a discordant response with only either detectable humoral or cellular response. Clinical features of the two subgroups differed with regard to previous treatment lines, which seem to affect the humoral more than the T cell axis. CLL-targeted treatments as well the underlying diseases itself affect B cells and self-evidently impact the humoral response. Our findings encourage immunization of patients even at advanced disease stages or heavily pre-treated as a subgroup that may respond with the T cellular axis.

Two patients showed a particular strong T cell response: One had been vaccinated thrice and the other had received a heterologous boosting (Fig. 1D). Data from more patients will need to prove if a booster vaccination is more likely to induce T cell response. Our data emphasize the importance of assessing the T cell response in patients with a limited serologic response. The best vaccination regime to promote those key players remains to be investigated. While heterologous immunization appears to elicit stronger T cell responses than homologous immunization [9], the chronological order for immunocompromised patients is unclear and needs further study.

A limitation of this study is the small sample size and the younger age of the control group (as compared with the SLL/CLL patients), as older individuals respond with lower antibody levels to vaccination. However, in the rather small fraction of SLL/CLL patients who responded to vaccination, similar titers of neutralizing antibodies were detectable in HC. Further, we only included one treatment-naïve patient and therefore cannot fully conclude the impact of CLL-directed treatment as compared with untreated CLL on cellular immunity. Previous trials reported inferior serologic immunogenicity in treatment-naïve patients as compared with patients previously treated [4, 5, 10]. Future studies should provide more data comparing those two subgroups of CLL patients and further focus on cellular immunity.

In conclusion, we demonstrate inferior T cell response to COVID-19 vaccines in SLL/CLL patients as compared with HC, supporting the importance of a third vaccine dose for those. The prime-boost regime, in particular the choice of best vaccine combination, is yet to determine. Our observation of discordant immune responses in the majority of patients indicates that the humoral response may not be reliable as the sole surrogate marker of protection in the patients with CLL and further B cell depleting malignancies, at least if negative.

## Supplementary information

Supplement
