# Supplementary material for: SARS-CoV-2 specific cellular response following COVID-19 vaccination in patients with chronic lymphocytic leukemia
Source: Leukemia. 2021 Dec 22;36(2):562–5. doi: 10.1038/s41375-021-01500-1 (PMC8693592; doi:10.1038/s41375-021-01500-1)
Supplement: Supplementary file 1 — Supplement [file 41375_2021_1500_MOESM1_ESM.docx]

**Supplemental Table 1**. Vaccination schemes in CLL patients and healthy volunteers.

| **Type of vaccination** |  | **Patients with CLL** | **Healthy control population** |
| --- | --- | --- | --- |
| **Analysis population, N** | | 23 | 12 |
|  | | | |
| **Sequence of vaccination, N (%)** | |  |  |
| AZD│AZD |  | 2 (8.7) | 1 (8.3) |
| AZD│BNT |  | 2 (8.7) | 1 (8.3) |
| BNT│BNT |  | 18 (78.3) | 10 (83.3) |
| BNT│BNT│BNT |  | 1 (4.3) | 0 (0.0) |

**Supplemental Table 2.** Humoral Responses by subgroups

| **Parameters N (%)** | | | **Patients with CLL (N=21)** | | |
| --- | --- | --- | --- | --- | --- |
|  |  |  | **Humoral response negative** | | **Humoral response positive** |
| **Humoral response** with detection of anti- SARS-CoV-2 spike-RBD IgG ≥ 7.1 BAU/ml, N (%) | | |  | 13 (61.9) | 8 (38.1) |
| **Age, median (range)** (years) | | |  | 71.0 (48-79) | 67.5 (49-77) |
| **Age group** (years) | | |  |  |  |
|  | >65 |  |  | 9 (69.2) | 4 (50.0) |
|  | >70 |  |  | 7 (53.8) | 4 (50.0) |
| **Male sex** | | |  | 10 (76.9) | 8 (100.0) |
| **Disease / treatment status** | | |  |  |  |
|  | Treatment-naïve |  |  | 0 (0.0) | 1 (14.3) |
|  | Previously treated |  |  | 13 (100.0) | 7 (87.5) |
| **Treatment prior vaccination** | | |  |  |  |
|  | Line of treatment, median (range) | |  | 2 (1-8) | 2 (1-5) |
|  |  | 1^st^ line |  | 2 (15.4) | 3 (42.9) |
|  |  | >1^st^ line |  | 11 (84.6) | 4 (50.0) |
|  | Treatment < 12 months prior vaccination | |  | 8 (61.5) | 1 (12.5) |
|  |  | without anti CD20^1^ |  | 3 (37.5) | 0 (0.0) |
|  |  | with anti CD20^2^ |  | 5 (62.6) | 1 (100.0) |
| **Type according to hierarchical model**† | | |  |  |  |
|  | del(17p) |  |  | 4 (30.8) | 0 (0.0) |
|  | del(11q) |  |  | 2 (15.4) | 3 (50.0) |
|  | Trisomy 12 |  |  | 1 (7.7) | 1 (16.7) |
|  | No abnormalities |  |  | 0 (0.0) | 1 (16.7) |
|  | del(13q) [single] |  |  | 6 (46.2) | 1 (16.7) |
| **IGHV mutational status** | | |  |  |  |
|  | Unmutated |  |  | 8 (72.7) | 4 (80.0) |
|  | Mutated |  |  | 3 (27.3) | 1 (20.0) |
| **TP53 mutational status** | | |  |  |  |
|  | Mutated |  |  | 9 (81.8) | 6 (100.0) |
|  | Unmutated |  |  | 2 (18.2) | 0 (0.0) |

† Cytogenetic subgroups were determined according to the hierarchical model of Döhner et al.^1^

^1^ Obinutuzumab, Obinutuzumab/Venetoclax; ^2^ Acalabrutinib, Acalabrutinib/Obinutuzumab, Acalabrutinib/Obinutuzumab/Venetoclax, Ibrutinib

**Supplemental Table 3**. T Cell Responses by subgroups

| **Parameters N (%)** | | | **Patients with CLL (N=21)** | | |
| --- | --- | --- | --- | --- | --- |
|  |  |  | **Cellular response negative** | | **Cellular response positive** |
| **Cellular response** with detection of SARS-CoV-2 specific T cells > 48 SFC/10^6^ PBMC, N (%) | | |  | 13 (61.9) | 8 (38.1) |
| **Age, median (range)** (years) | | |  | 72 (48-79) | 63 (49-79) |
| **Age group** (years) | | |  |  |  |
|  | >65 |  |  | 10 (76.9) | 3 (37.5) |
|  | >70 |  |  | 8 (61.5) | 3 (37.5) |
| **Male sex** | | |  | 11 (84.6) | 7 (87.5) |
| **Disease / treatment status** | | |  |  |  |
|  | Treatment-naïve |  |  | 1 (7.7) | 0 (0.0) |
|  | Previously treated |  |  | 12 (92.3) | 8 (100.0) |
| **Treatment prior vaccination** | | |  |  |  |
|  | Line of treatment, median (range) | |  | 2 (1-8) | 2 (1-5) |
|  |  | 1^st^ line |  | 4 (33.3) | 1 (12.5) |
|  |  | >1^st^ line |  | 8 (61.5) | 7 (87.5) |
|  | Treatment < 12 months prior vaccination | |  | 4 (33.3) | 4 (50.0) |
|  |  | without anti CD20^1^ |  | 3 (75.0) | 0 (0.0) |
|  |  | with anti CD20^2^ |  | 1 (25.0) | 4 (100.0) |
| **Type according to hierarchical model**† | | |  |  |  |
|  | del(17p) |  |  | 3 (27.3) | 1 (12.5) |
|  | del(11q) |  |  | 2 (18.2) | 3 (37.5) |
|  | Trisomy 12 |  |  | 1 (9.1) | 1 (12.5) |
|  | No abnormalities |  |  | 1 (9.1) | 0 (0.0) |
|  | del(13q) [single] |  |  | 4 (36.4) | 3 (37.5) |
| **IGHV mutational status** | | |  |  |  |
|  | Unmutated |  |  | 8 (72.7) | 4 (80.0) |
|  | Mutated |  |  | 3 (27.3) | 1 (20.0) |
| **TP53 mutational status** | | |  |  |  |
|  | Mutated |  |  | 8 (80.0) | 7 (100.0) |
|  | Unmutated |  |  | 2 (20.0) | 0 (0.0) |

† Cytogenetic subgroups were determined according to the hierarchical model of Döhner et al.^1^

^1^ Obinutuzumab, Obinutuzumab/Venetoclax; ^2^ Acalabrutinib, Acalabrutinib/Obinutuzumab, Acalabrutinib/Obinutuzumab/Venetoclax, Ibrutinib

**References**

1. Döhner H, Stilgenbauer S, Benner A, et al. Genomic aberrations and survival in chronic lymphocytic leukemia. *N Engl J Med*. 2000;343(26):1910-1916.
